# Phosphorus restriction does not prevent the increase in fibroblast growth factor 23 elicited by high fat diet

**DOI:** 10.1371/journal.pone.0198481

**Published:** 2018-06-01

**Authors:** Rafael Rios, Carmen Pineda, Ignacio Lopez, Juan Muñoz-Castañeda, Mariano Rodriguez, Escolastico Aguilera-Tejero, Ana I. Raya

**Affiliations:** 1 Department of Animal Medicine and Surgery, University of Cordoba, Campus Universitario Rabanales, Cordoba, Spain; 2 Maimonides Biomedical Research Institute of Cordoba (IMIBIC), Reina Sofia University Hospital, University of Cordoba, Cordoba, Spain; Max Delbruck Centrum fur Molekulare Medizin Berlin Buch, GERMANY

## Abstract

This study was designed to evaluate the influence of phosphorus (P) restriction on the deleterious effects of high fat diets on mineral metabolism. Twenty-four rats were allotted to 3 groups (n = 8 each) that were fed different diets for 7 months. Rats in group 1 were fed normal fat-normal P (0.6%) diet (NF-NP), rats in group 2 were fed high fat- normal P diet (HF-NP) and rats in group 3 were fed high fat-low P (0.2%) diet (HF-LP). Blood, urine and tissues were collected at the end of the experiments. When compared with the control group (NF-NP), rats fed HF diets showed increases in body weight, and in plasma concentrations of triglycerides and leptin, and decreased plasma calcitriol concentrations. In rats fed HF-NP plasma fibroblast growth factor 23 (FGF23) was higher (279.6±39.4 pg/ml vs 160.6±25.0 pg/ml, p = 0.018) and renal klotho (ratio klotho/GAPDH) was lower (0.75±0.06 vs 1.06±0.08, p<0.01) than in rats fed NF-NP. Phosphorus restriction did not normalize plasma FGF23 or renal klotho; in fact, rats fed HF-LP, that only ingested an average of 22.9 mg/day of P, had higher FGF23 (214.7±32.4 pg/ml) concentrations than rats fed NF-NP (160.6±25.0 pg/ml), that ingested and average of 74.4 mg/day of P over a 7 month period. In conclusion, our results demonstrate that severe P restriction over a prolonged period of time (7 months) does not normalize the increase in circulating FGF23 induced by HF diets. These data indicate that the deleterious effects of high fat diet on the FGF23/klotho axis are not eliminated by reduced P intake.

## Introduction

Obesity and its related metabolic complications represent a major health concern, not only due to their influence on morbidity and mortality but also for the high healthcare cost associated to this disease [1,2]. A major factor in the development of obesity is excessive caloric intake which is favored by the ingestion of energy-dense processed foods (fast food). In addition to their high caloric concentration, processed foods are often rich in phosphate (P) [3]. Phosphate is a common food additive and the inorganic P added to processed foods is more readily absorbed than organic P [4]. Moreover, the high fat content of many fast foods also enhances intestinal P absorption [5]. Therefore, individuals eating fast food often ingest an overload of P that must be excreted by the kidney to maintain P homeostasis.

Fibroblast growth factor 23 (FGF23) is a major phosphaturic hormone. In the kidney, FGF23 promotes P excretion after binding to its receptor (FGFR1) and co-receptor (klotho) [6]. Increased levels of FGF23, which are usually found in hyperphosphatemic uremic patients, have been reported as a risk factor of cardiovascular mortality [7]. The relationship between high FGF23 and mortality is not restricted to patients with kidney disease but has also been identified in the general population [8,9].

An increase in circulating levels of FGF23 has been reported in rats fed high fat diets [10]. The mechanism for increased FGF23 production seems to be related to decreased expression of renal klotho after feeding high fat diet. Klotho down-regulation generates tubular resistance to FGF23 and thus more FGF23 is needed for P excretion [11]. Additionally, a recent study has shown that an excessive tubular load of P down-regulates the expression of klotho in the kidney. Renal klotho was reduced in rats with increased tubular load of P and was higher in uremic rats on a low P diet than in uremic rats in a high P diet [12].

We hypothesize that reducing P load by feeding a diet with low P content would prevent the decrease in renal klotho and the increase in FGF23 elicited by high fat diet. Thus the main objective of this study was to evaluate the influence of P restriction on the deleterious effects of high fat diets on mineral metabolism. Additionally, since previous studies [10] were of short duration (1 month), a secondary objective was to evaluate the influence of high fat diets on FGF23 over a more extended period of time (7 months).

## Material and methods

### Ethics

All experimental protocols were reviewed and approved by the Ethics Committee for Animal Research of the University of Cordoba (Cordoba, Spain). All protocols were carried out in accordance with the approved guidelines. They followed the guiding principle laid down by the Higher Council of Scientific Research of Spain following the normal procedures directing animal welfare (Real Decreto 223/88, BOE of 18 of March) and adhered to the recommendations included in the Guide for Care and Use of Laboratory Animals (US Department of Health and Human Services, NIH) and European laws and regulations on protection of animals, under the advice of specialized personnel.

### Animals and diets

Twenty-four 2 months-old Wistar rats, provided by the Animal Housing Facilities of the University of Cordoba (Cordoba, Spain), were housed with a 12h/12h light/dark cycle. Appropriate measures were taken to ensure animal welfare and to address the basic behavioral and physiological needs of rats.

Two diets with normal P (NP = 0.6%) were used in the experiments: normal fat content diet (NF-NP) with a 5% fat concentration that provided Metabolizable Energy = 3518 kcal/kg (Altromin C 1090–10, AltrominSpezialfutter GmbH, Germany) and a high fat content diet (HF-NP) with a 35% fat concentration that provided Metabolizable Energy = 5241 kcal/kg (Altromin C 1090–60, AltrominSpezialfutter GmbH, Germany). In addition, a HF diet with low P (LP = 0.2%) was also used (HF-LP). All diets contained 0.6% of Ca and 500 IU/g of vitamin D.

### Experimental design

Rats were allotted to 3 groups (n = 8 each) which were fed ad libitum the study diets for 7 months. Rats in group 1 were fed NF-NP, rats in group 2 were fed HF-NP and rats in group 3 were fed HF-LP. During the last week of the trial the rats were housed in metabolic cages, allowing daily control of food and water intake and collection of urine. At the end of the experiment, rats were sacrificed by exsanguination under general anesthesia (inhaled sevoflurane) to obtain blood and tissue samples.

### Blood chemistries

Blood was collected from the abdominal aorta at the time of sacrifice. Blood glucose was measured immediately after collection with a blood glucose meter (Bayer Consumer Care AG, Basel, Switzerland). Afterwards, plasma was separated by centrifugation and stored at –20°C until assayed. Plasma concentrations of total cholesterol, triglycerides, urea, creatinine, calcium and phosphorus were measured by spectrophotometry (BioSystems SA, Barcelona, Spain). ELISA tests were used to quantify plasma intact FGF23 (Immutopics, San Clemente, CA), leptin (Millipore Corporation, Billerica, MA, USA), tumor necrosis factor alpha (TNFα) (eBioscience, Bender MedSystems GmbH, Vienna, Austria) and parathyroid hormone (PTH) (Immutopics, San Clemente, CA). Radioimmunoassay (Immunodiagnostic Systems Ltd, Boldon, UK) was used in plasma samples to determine 1,25-dihydroxyvitamin D (calcitriol).

### Urine chemistries

Urine phosphorus concentrations were measured by spectrophotometry (BioSystems SA, Barcelona, Spain).

### Real-Time-Polymerase Chain Reaction (RT-PCR)

Analyses of renal klotho mRNA were performed by quantitative Real-Time PCR. Kidney tissue was disrupted using liquid nitrogen and grinded thoroughly with a mortar. Renal total RNA was extracted with using chloroform and isopropanol precipitation method and a treatment with DNAse I amplification Grade (Sigma-Aldrich) and quantified by spectrophotometry (ND-1000, Nanodrop Technologies, Wilmington, DE). Fifty ng of total RNA were used to analyze mRNA expression in the Light Cycler thermal cycler system (Roche Diagnostics, Indianapolis, IN, USA). RT-PCR was performed in one step, with the QuantiTect SYBR Green RT-PCR kit (Qiagen GmbH, Hilden, Germany) according to the manufacturer’s protocol. Rat primers for membrane Klotho were designed with the free Oligo 7 software (http://www.oligo.net/), and the sequence is F: 5´-CTCTGAAAGCCTACGTGTTGG-3´ and R: 5´-TAGAAACGAGATGAAGGCCAG-3´. Results were normalized to that of GAPDH. Primers sequence for GAPDH is F: 5´-AGGGCTGCCTTCTCTTGTGAC-3´ and R: 5´-TGGGTAGAATCATACTGGAACATGTAG-3´. Quantification of relative expression was determined by the 2(2ΔCt) method.

### Protein extraction and Western blot analysis

Proteins were isolated from renal tissue by using a lysis buffer containing HEPES (10 mmol/l), KCl (10 mmol/l), EDTA (0.1 mmol/l), EGTA (0.1 mmol/l), DTT (1 mmol/l), PMSF (0.5 mmol/l), protease inhibitor cocktail (70 μg/ml), and I-Gepal CA-630 (0.6%), pH 7.9 (Sigma Aldrich, St. Louis, MO). Protein concentration was determined by Bradford method. For Western Blot analysis, 50 μg of protein (klotho) or 100 μg (NaPiIIa) of protein were electrophoresed on a 10% SDS-polyacrilamide gel (Invitrogen, Carlsbad, CA) and electrophoretically transferred (Transfer Systems, BioRad, Hercules, CA) from the gels onto nitrocellulose membranes (Invitrogen). The following steps were performed with gentle shaking. Membranes were incubated in TTBS-L solution [20 mM Tris-HCl (pH 7.6), 0.2% Tween 20, 150 mM NaCl] (Sigma Aldrich), and 5% nonfat dry milk (Bio-Rad) at room temperature for 1 hour to avoid nonspecific binding. Membranes were then washed with TTBS buffer (the same composition as TTBS-L without nonfat dry milk) and incubated overnight at 4°C temperature with a rat anti-klotho antibody (Alpha Diagnostic Int, San Antonio, TX; 0.5 μg/ml) or a rabbit polyclonal anti-NaPiIIa antibody (Abcam plc, Cambridge, UK; 1 μg/ml). The membranes were then washed with TTBS buffer and immunolabeled using a peroxidase-conjugated secondary antibody (1:5000 dilution; Santa Cruz Biotechnology Inc, Santa Cruz, CA). Finally, they were revealed on autoradiographic film using ECL Plus Western Blotting Detection System (GE Healthcare, Piscataway, NJ). GAPDH was used as housekeeping protein to ensure equal loading of the gels. Protein levels were quantified using ImageJ software (National Institutes of Health, Bethesda, MD).

### Renal histopathology

Kidney tissue samples were fixed in 10% buffered formalin, embedded in paraffin, sectioned and processed for staining with hematoxylin and eosin (H&E), periodic acid-Schiff (PAS), Masson’s Trichrome and Von Kossa stains. Lesions were scored using a semi quantitative scale graded from 0–3: 0 (absent), 1 (slight), 2 (moderate) or 3 (severe). This scale was constructed by a previous scanning of all the samples under study in which the more severe lesions were identified and were assigned a value = 3. For statistical analysis lesions were grouped into three categories: a) glomerular lesions (glomerular retraction and sclerosis), b) tubular lesions (tubular atrophy, tubular hyperplasia and thickening of basement membrane) and c) interstitial lesions (interstitial edema and inflammatory infiltrate). Analyses were performed in a blind manner.

### Statistics

Values are expressed as the mean ± standard error (SE). The difference between means for the three experimental groups was assessed by ANOVA. Fisher LSD test was used as a post-hoc procedure. A correlation study was carried out using the Pearson test. p<0.05 was considered significant.

## Results

**Fig 1:**
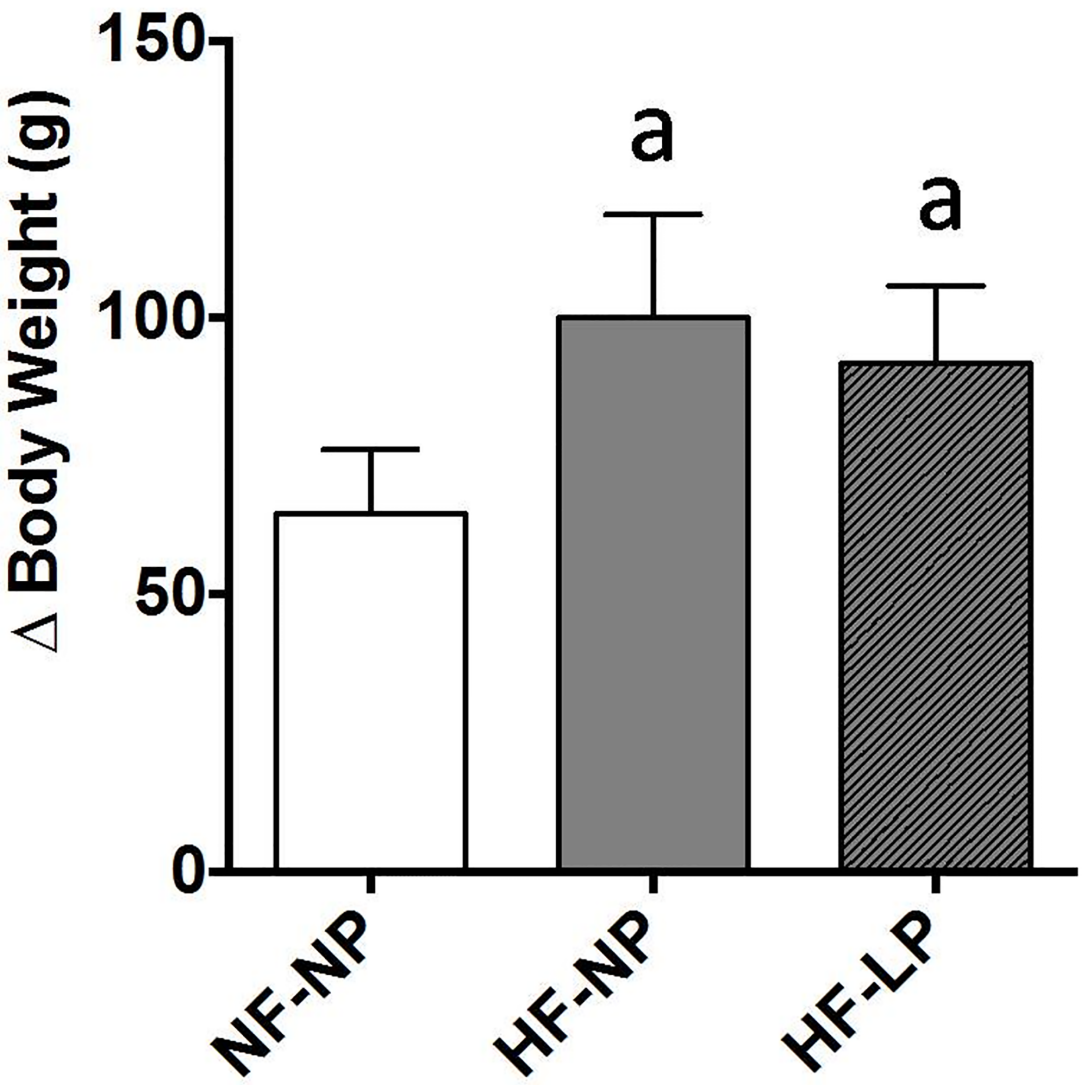
Body weight. Increase in body weight after the 7 months that lasted the experiments in rats (n = 8 per group) fed diets with normal fat and normal phosphorus (NF-NP), high fat and normal phosphorus (HF-NP) and high fat and low phosphorus (HF-LP). ^a^p<0.05 vs NF-NP.

At the beginning of the study all rats had similar body weight that ranged between 239.4±1.7 and 251.2±3.3 g. During the 7 months that lasted the experiment rats experienced an increase in body weight that was more accentuated in the groups fed a HF diet. Phosphorus restriction attenuated the increase in body weight in rats fed HF, but the differences were small and non-significant (Fig 1). Mean daily intake of P (mg/day) was not influenced by the amount of ingested fat but, obviously, was much lower (p<0.001) in the P restricted group: NF-NP = 74.4±6.3, HF-NP = 77.9±3.7, HF-LP = 22.9±1.4.

Blood glucose and cholesterol concentrations were not different between groups. However, plasma triglycerides were higher in rats fed HF than in rats fed NF: HF-NP = 0.94±0.1 mmol/l vs NF-NP = 0.58±0.1 mmol/l, p = 0.002. Phosphorus restriction led to a non-significant decrease in triglyceride concentrations in HF rats. Plasma leptin concentrations were also higher (p<0.05) in rats fed HF (5.2±0.8 and 5.8±0.5 ng/ml) than in rats fed NF (3.9±0.5 ng/ml) and were not influenced by P restriction; these data are depicted in Table 1.

**Table 1:** Blood biochemistry. Plasma concentrations of parameters related to energy metabolism, mineral metabolism, renal function, and inflammation in rats fed diets with normal fat and normal phosphorus (NF-NP), high fat and normal phosphorus (HF-NP) and high fat and low phosphorus (HF-LP).

	NF-NP (n = 8)	HF-NP (n = 8)	HF-LP (n = 8)
**Glucose (mmol/l)**	6.43 ± 0.3	5.54 ± 0.3	5.76 ± 0.2
**Total Cholesterol (mmol/l)**	1.31 ± 0.2	1.28 ± 0.2	1.37 ± 0.1
**Triglycerides (mmol/l)**	0.58 ± 0.1	0.94 ± 0.1^a^	0.63 ± 0.0
**Leptin (ng/ml)**	3.9 ± 0.5	5.2 ± 0.8^a^	5.8 ± 0.5^a^
**Calcium (mmol/l)**	2.3 ± 0.1	2.2 ± 0.1	2.2 ± 0.1
**Phosphorus (mmol/l)**	1.2 ± 0.1	1.0 ± 0.1	1.0 ± 0.1
**PTH (pmol/l)**	29.8 ± 6.5	27.5 ± 6.0	21.0 ± 1.6
**Calcitriol (pmol/l)**	50.7 ± 21.5	5.3 ± 0.9^a^	12.3 ± 1.9^a^^b^
**Urea (mmol/l)**	3.98 ± 0.51	4.98 ± 0.54	4.99 ± 0.44
**Creatinine (μmol/l)**	65 ± 4	73 ± 3	81 ± 2
**TNFα (pg/ml)**	62.7 ± 4.6	75.5 ± 7.6	77.7 ± 4.5

^a^p<0.05 vs NF-NP,

^b^p<0.05 vs HF-NP.

Plasma Ca concentrations were not different in the study groups. Plasma P was not influenced by the caloric content of the diet: NF-NP = 1.2±0.1 mmol/l vs HF-NP = 1.0±0.1 mmol/l. Phosphorus restriction did not modify plasma P in rats fed HF (1.0±0.1 mmol/l). Plasma PTH was not different in HF vs NF groups but tended to be decreased in the HF-LP group. Plasma calcitriol concentrations were higher in rats fed normal fat: NF-NP = 50.7±21.5 pmol/l vs HF-NP = 5.3±0.9 pmol/l, p<0.05. Phosphorus restriction led to a modest increase in plasma calcitriol in rats fed high fat, HF-LP = 12.3±1.9 pmol/l, p<0.05 vs HF-NP. No significant differences in either urea or creatinine were observed between the experimental groups, although rats fed HF had slightly higher values of both parameters. Plasma concentration of TNFα tended to be higher in rats fed HF (75.5±7.6 and 77.7±4.5 pg/ml) when compared with rats fed NF (62.7±4.6 pg/ml) but differences were not significant (Table 1).

**Fig 2:**
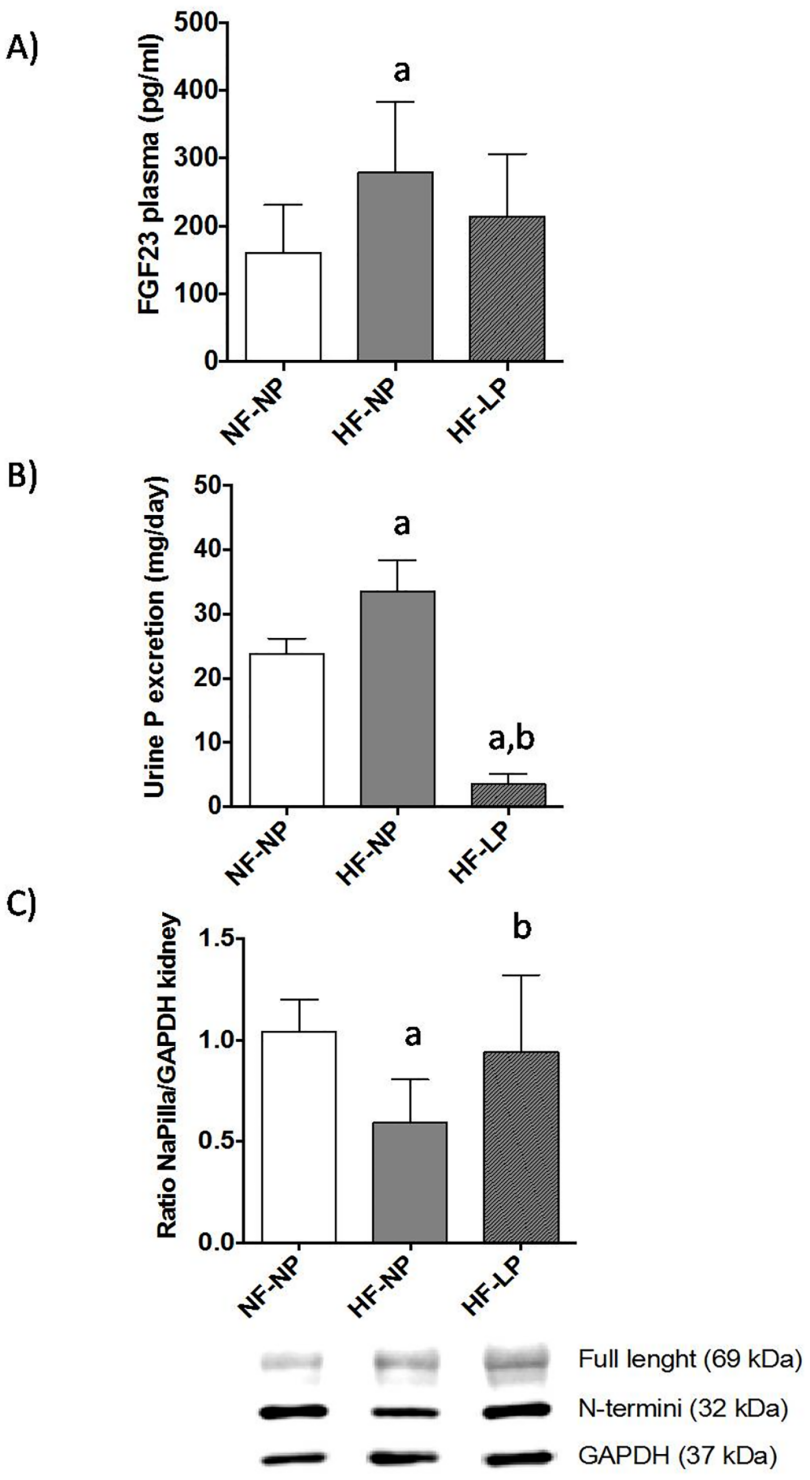
Plasma FGF23, urinary excretion of P and renal NaPiIIa. **(A)** Circulating concentrations of FGF23 in rats (n = 8 per group) fed diets with normal fat and normal phosphorus (NF-NP), high fat and normal phosphorus (HF-NP) and high fat and low phosphorus (HF-LP). (B) Urinary excretion of P (mg/day) in rats (n = 8 per group) fed diets with normal fat and normal phosphorus (NF-NP), high fat and normal phosphorus (HF-NP) and high fat and low phosphorus (HF-LP). (C) Expression of NaPiIIa in the kidneys of rats (n = 8 per group) fed diets with normal fat and normal phosphorus (NF-NP), high fat and normal phosphorus (HF-NP) and high fat and low phosphorus (HF-LP). The original gel from which blots have been extracted is shown in S1 Fig. ^a^p<0.05 vs NF-NP, ^b^p<0.05 vs HF-NP.

Plasma concentrations of FGF23 were higher in the HF groups but significant differences were only found between HF-NP, 279.6±39.4 pg/ml and NF-NP, 160.6±25.0 pg/ml (p = 0.018). A non-significant tendency to decreased FGF23 was detected in P restricted rats (Fig 2A).

**Fig 3:**
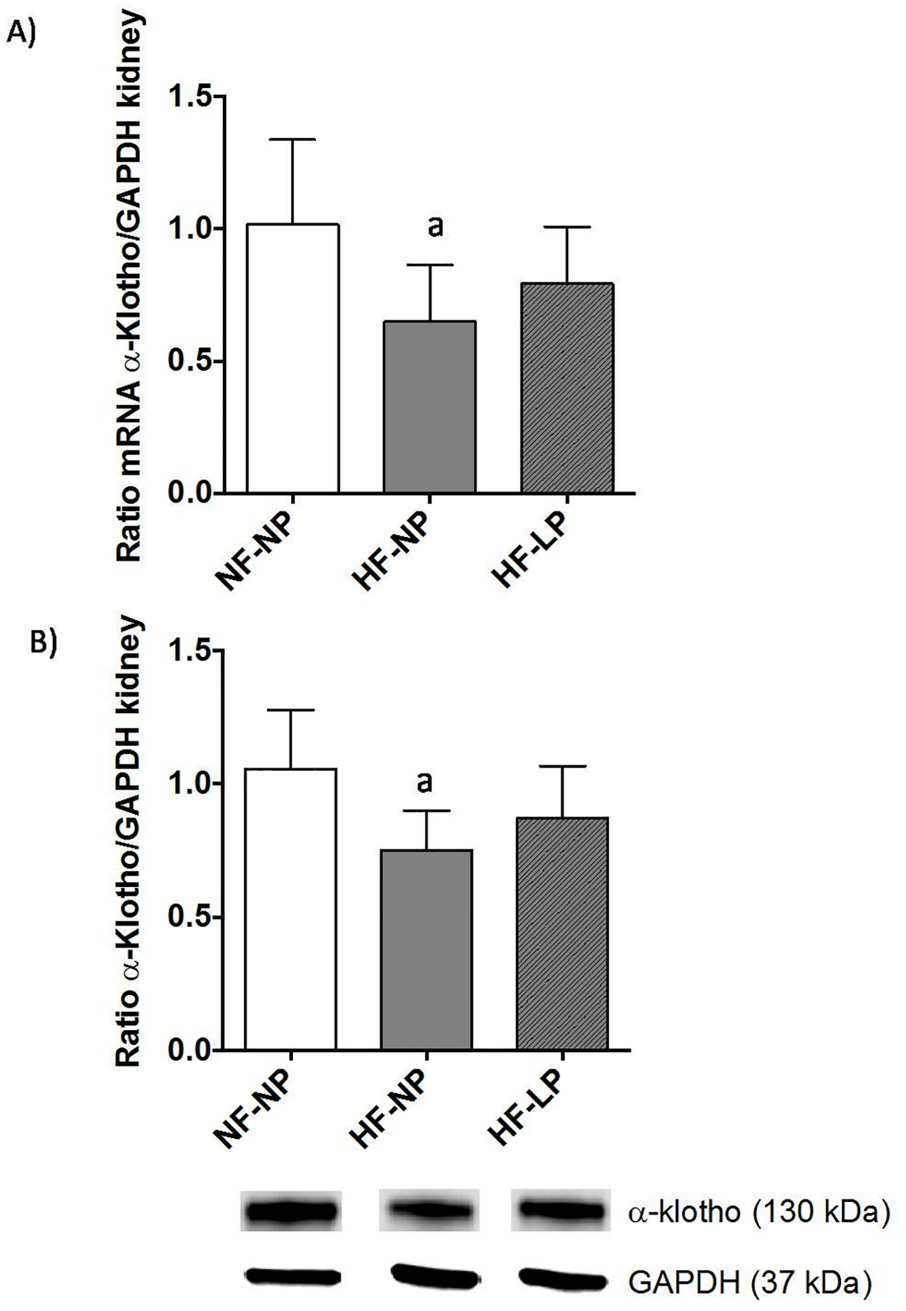
Renal klotho. Klotho mRNA (A) and protein (B) in the kidneys of rats (n = 8 per group) fed diets with normal fat and normal phosphorus (NF-NP), high fat and normal phosphorus (HF-NP) and high fat and low phosphorus (HF-LP). The original gel from which blots have been extracted is shown in S2 Fig. ^a^p<0.05 vs NF-NP.

Renal klotho, both at the mRNA and protein levels, changed in accordance, but in opposite direction, to FGF23. Thus, mRNA (ratio klotho/GAPDH) was lower (p<0.01) in rats fed HF-NP (0.65±0.09) than in rats fed NF-NP (1.06±0.13). A significant (p<0.01) decrease in renal klotho protein (ratio klotho/GAPDH) was also found in the HF-NP group (0.75±0.06) when compared with the NF-NP group (1.06±0.08) (Fig 3).

Urinary excretion of P was higher (p<0.01) in rats fed HF-NP (33.5±1.7 mg/day) than in rats fed NF-NP (23.8±0.8 mg/day). Rats fed HF-LP excreted very little P (3.5±0.6) mg/day, p<0.01 vs NP groups (Fig 2B). Renal NaPiIIa (ratio NaPiIIa/GAPDH) was decreased (p<0.01) in rats fed HF-NP (0.59±0.05) when compared with rats fed NF-NP (1.04±0.04). Phosphorus restriction restored NaPiIIa expression in rats fed HF to values (0.94±0.09) than were not different from rats fed NF (Fig 2C).

**Fig 4:**
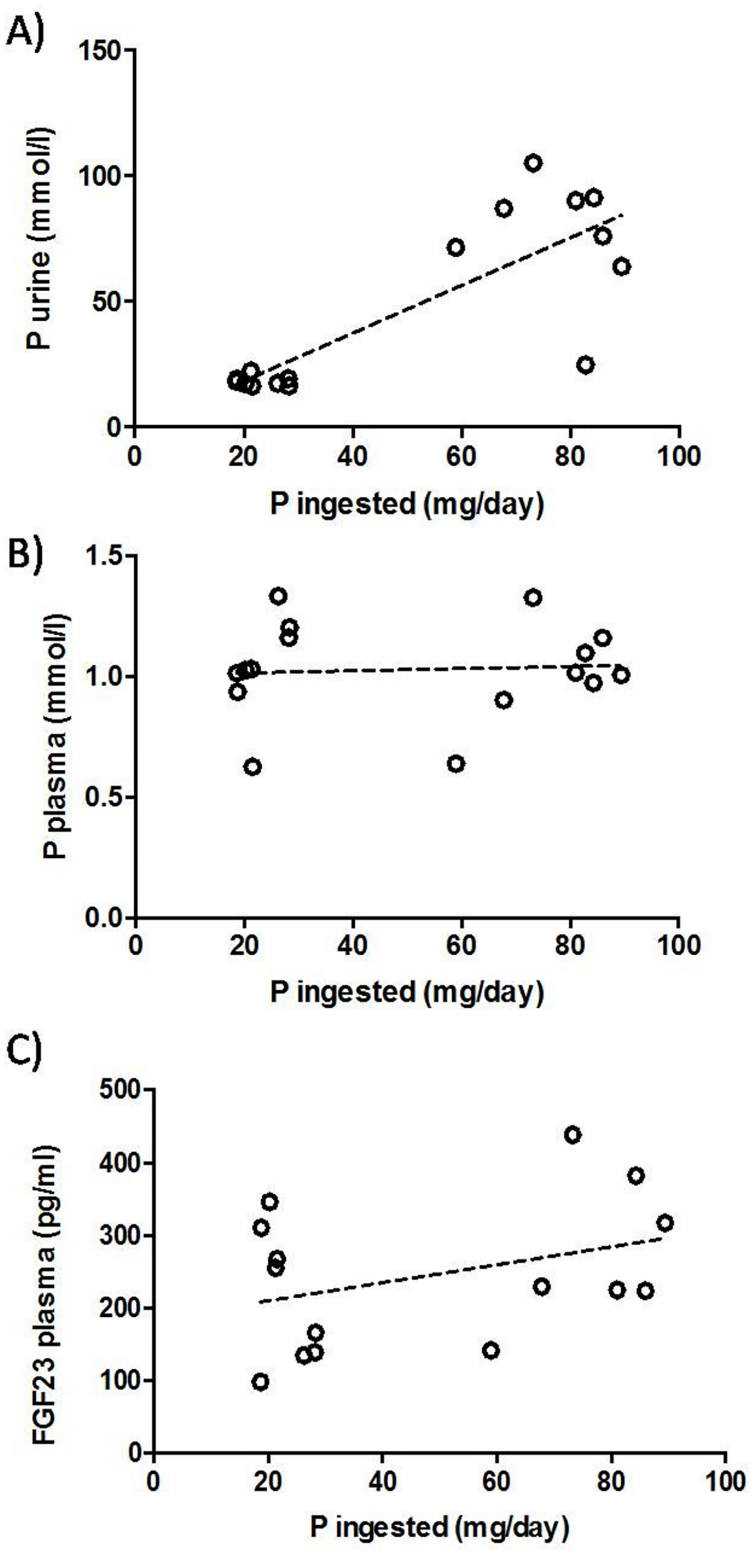
Diet vs urine & plasma P and FGF23. Correlation between P ingested and (A) P urine (r^2^ = 0.663, p<0.001), (B) P plasma (r^2^ = 0.004, p = 0.806), and (C) FGF23 plasma (r^2^ = 0.130, p = 0.187) in rats fed high fat diets with normal and low phosphorus content.

In rats fed HF, the amount of P ingested was well correlated (r^2^ = 0.663) with urinary excretion of P (Fig 4A). However, no significant correlation was found between ingested P and either plasma P or FGF23 (Fig 4B and 4C).

Histopathological studies demonstrated the presence of subtle renal lesions in rats fed HF diets. The number of retracted and sclerotic glomeruli was significantly higher in rats fed HF and P restriction did not influence glomerular damage. Similarly, rats fed HF showed more tubular atrophy and hyperplasia, as well as thickening of the basement membrane than rats fed NF. Phosphorus restriction tended to attenuate tubular lesions but the differences were not significant. Interstitial edema and infiltrate were also more prominent in rats fed HF and, in this case, P restriction significantly attenuated interstitial pathology (Table 2). Neither fibrosis nor nephrocalcinosis were detected in any experimental group.

**Table 2:** Renal histopathology. Glomerular retraction and sclerosis (Glomerular lesions); tubular atrophy, hyperplasia and thickening of basement membrane (Tubular lesions); and interstitial edema and infiltrate (Interstitial lesions) in rats fed diets with normal fat and normal phosphorus (NF-NP), high fat and normal phosphorus (HF-NP) and high fat and low phosphorus (HF-LP).

	NF-NP (n = 8)	HF-NP (n = 8)	HF-LP (n = 8)
**Glomerular lesions**	0.5 ± 0.1	1.1 ± 0.3^a^	1.3 ± 0.4^a^
**Tubular lesions**	0.1 ± 0.1	1.2 ± 0.1^a^	0.9 ± 0.2^a^
**Interstitial lesions**	0.4 ± 0.1	0.8 ± 0.1^a^	0.5 ± 0.1^b^

Semiquantitative scale (0–3).

^a^p<0.05 vs NF-NP,

^b^p<0.05 vs HF-NP.

## Discussion

This study was designed to investigate the effect of restricting P intake on the changes in mineral metabolism elicited by HF diets. Our results demonstrate that marked P restriction over a prolonged period of time (7 months) does not normalize the elevated circulating FGF23 levels induced by HF diets. These data indicate that the deleterious effects of high fat diet on the FGF23/klotho axis are not eliminated by reduced P intake.

The increase in plasma concentrations of FGF23 detected in the present study after feeding HF diets confirms previous results which were obtained over a much shorter period of time (1 month) [10]. It is interesting to note that the magnitude of changes in both plasma FGF23 and renal klotho was similar after 1 and 7 months of exposure to HF diets. Thus the effect of HF diets on these parameters does not seem to be progressive.

As it has been previously reported, the mechanism for increased FGF23 in rats fed HF diets is likely related to the decrease in renal klotho. Renal klotho has been shown to decrease in response to HF diets both in Wistar rats [10] and in APoE knockout mice [13]. When renal klotho is decreased, tubular resistance to FGF23 action ensues and more FGF23 is needed to maintain phosphaturia, consequently resulting in an increase in circulating levels of FGF23 [11]. In addition, feeding high fat and obesity may elicit systemic inflammation [14] and renal injury [15] which could also influence FGF23 [11]. In the present study, a tendency to increased TNFα and minor renal lesions were observed in rats fed HF. Therefore, in addition to the reduction in renal klotho, both inflammation and renal damage may have played a marginal role in the increase in FGF23.

Based on recent published data that demonstrated that klotho expression in the kidney is regulated by the P load in the renal tubule [12], it was hypothesized that reducing P intake, by feeding a 0.2% P diet, would restore renal klotho and decrease circulating FGF23 levels in rats fed HF. Our results show that P restriction resulted in a discrete increase in renal klotho expression in rats fed HF diets. A small and non-significant decrease in circulating levels of FGF23 was also observed in rats fed HF diets after P restriction. These results support previous data on the role of tubular P load on klotho but, on the other hand, demonstrate that P restriction is not able to fully compensate the changes in FGF23 elicited by feeding a HF diet. In fact, in this model the stimulatory effect of HF diet on FGF23 overcame the inhibitory effect of P restriction and the present data point towards a preferential regulation of FGF23 by fat intake rather than by P intake, as demonstrated by direct comparison of HF-LP and NF-NP groups. This contention is illustrated by the fact that rats fed HF-LP, that only ingested an average of 22.9 mg/day of P, had higher FGF23 concentrations than rats fed NF-NP, that ingested and average of 74.4 mg/day of P over a 7 month period.

The main physiologic role of FGF23 is to enhance phosphaturia [6] and this is accomplished by down-regulating P transporters, mainly NaPiIIa, in the renal tubule. The reduction of NaPiIIa restricts P reabsorption and promotes urinary excretion of P [16]. In our study, NaPiIIa was regulated in accordance to the changes in FGF23. Therefore, feeding HF resulted in a significant decrease in NaPiIIa and increased urinary excretion of P. Interestingly, in rats fed HF, P restriction increased NaPiIIa to almost to the same level than in rats fed NF, contrary to what was observed with FGF23. These data are in agreement with previous reports suggesting that, in addition to FGF23, NaPiIIa may be directly regulated by P [17].

It is surprising that high fat intake has such a profound influence on FGF23. It remains to be determined to what point FGF23 regulation by fat intake is an epiphenomenon or a reflection of further, and as yet not clarified, physiologic actions of FGF23. FGF23 plays a pivotal role in mineral metabolism and is consistently increased in patients with chronic kidney disease [18]. In addition, FGF23 has been reported to be increased in obese people [19] and a recent study identified energy intake as a potential predictor of plasma FGF23 concentrations [20]. In this context it is relevant to point out that klotho has been shown to be implicated in energy metabolism [21,22].

Parameters related to energy intake may influence the increase in FGF23 after feeding HF diets. One factor that might be relevant is the increase in plasma leptin concentrations observed in rats fed HF. Leptin has been reported to stimulate FGF23 secretion by osteocytes [23]. The effect of leptin is mediated by up-regulation of the stimulatory action of calcitriol on skeletal synthesis of FGF23 [24]. However, in the present study, in agreement with previous data [10,25], calcitriol levels were very low in rats fed HF diets deeming unlikely a leptin-mediated stimulatory effect of calcitriol as responsible for the increase in FGF23. Actually, since FGF23 is known to decrease calcitriol synthesis through inhibition of 1-alpha-hydroxylase activity in the kidney [26], it seems more likely that the high FGF23 may be responsible for the low calcitriol concentrations in rats fed HF diets.

A remarkable finding of this study is that plasma concentrations of P did not change in rats fed different amounts of P. Plasma P was maintained constant by adjusting urinary excretion of P which showed an excellent correlation with P ingestion. However, it is interesting to note that FGF23 had a poor correlation with ingested P and that many rats fed HF-LP had high FGF23 concentrations, even though their urinary excretion of P was consistently low. This finding also points toward a regulation of FGF23 by high fat diet, independent of P intake.

In conclusion, the results of this study indicate that dietary P restriction did not prevent the increase in FGF23 elicited by feeding HF diets.

## Supporting information

S1 Fig Renal NaPiIIa Western blot. Original Western blot gel from which the immunoblots shown in Fig 2 were extracted.
(DOCX)

S2 Fig Renal klotho Western blot. Original Western blot gel from which the immunoblots shown in Fig 3 were extracted.
(DOCX)
