# Supplementary material for: Phosphorus restriction does not prevent the increase in fibroblast growth factor 23 elicited by high fat diet
Source: PLoS One. 2018 Jun 1;13(6):e0198481. doi: 10.1371/journal.pone.0198481 (PMC5983526; doi:10.1371/journal.pone.0198481)
Supplement: Supplementary file 2 [file pone.0198481.s002.docx]

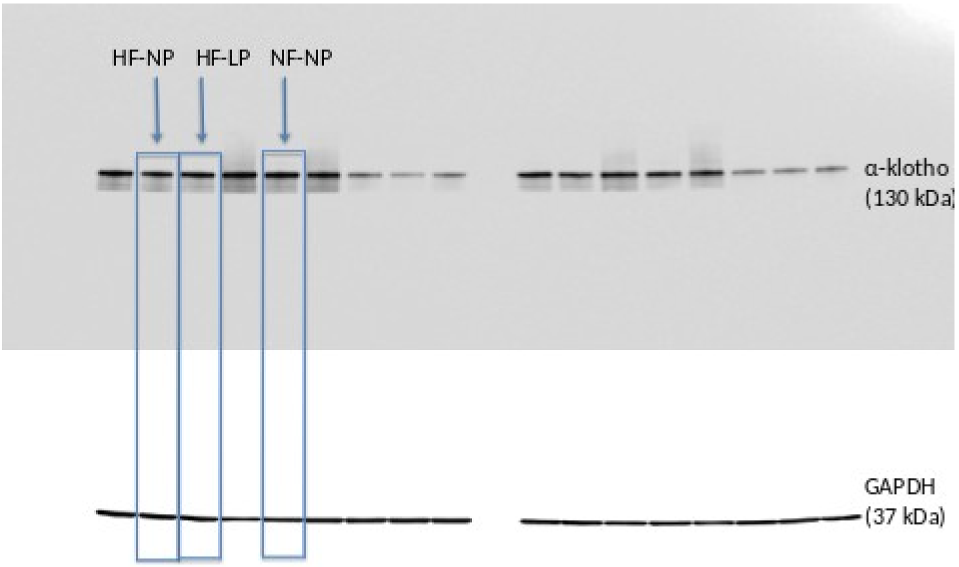
HF-NP
HF-LP
NF-NP
α-klotho
(130 kDa)
GAPDH
(37 kDa)
